# Systemic Infection through the Pharyngeal Lymphoid Ring Calling for Surgical Intervention

**Published:** 1912-11

**Authors:** 


Systemic Infection Through the Pharyngeal Lymphoid Ring Calling for Surgical Intervention.—C. B. Wylie of Chattanooga (*Jour. Oph. and Oto-Laryn.*, Aug., 1912.) details the anatomy of the pharyngeal ring and describes the symptoms of lacunar inflammation. The complications are much more important than the local condition. There is no part of the human economy where the lymphatic system will so rapidly disseminate septic materials as the lymphatic ring of the pharynx. Clinical experience makes it possible and often quite probable that the source of various inflammations of the serous membranes, in which no other definite cause can be discovered, is to be sought for in this locality. Tuberculosis in different parts of the body, muscular and articular rheumatism, nephritis, and diabetes may have their beginnings here. The size of the tonsils have but little bearing upon the symptoms (other than mechanic.) Many times do we find pronounced local or constitutional symptoms where the tonsil is quite small.

Operation is advised and a method for removing the entire gland is given.

